# Budget Constraints Affect Male Rats’ Choices between Differently Priced Commodities

**DOI:** 10.1371/journal.pone.0129581

**Published:** 2015-06-08

**Authors:** Marijn van Wingerden, Christine Marx, Tobias Kalenscher

**Affiliations:** Comparative Psychology, Institute for Experimental Psychology, Heinrich-Heine University Düsseldorf, Universitätsstrasse 1, D-40225, Düsseldorf, Germany; Institutes for Behavior Resources and Johns Hopkins University School of Medicine, UNITED STATES

## Abstract

Demand theory can be applied to analyse how a human or animal consumer changes her selection of commodities within a certain budget in response to changes in price of those commodities. This change in consumption assessed over a range of prices is defined as demand elasticity. Previously, income-compensated and income-uncompensated price changes have been investigated using human and animal consumers, as demand theory predicts different elasticities for both conditions. However, in these studies, demand elasticity was only evaluated over the entirety of choices made from a budget. As compensating budgets changes the number of attainable commodities relative to uncompensated conditions, and thus the number of choices, it remained unclear whether budget compensation has a trivial effect on demand elasticity by simply sampling from a different total number of choices or has a direct effect on consumers’ sequential choice structure. If the budget context independently changes choices between commodities over and above price effects, this should become apparent when demand elasticity is assessed over choice sets of any reasonable size that are matched in choice opportunities between budget conditions. To gain more detailed insight in the sequential choice dynamics underlying differences in demand elasticity between budget conditions, we trained N=8 rat consumers to spend a daily budget by making a number of nosepokes to obtain two liquid commodities under different price regimes, in sessions with and without budget compensation. We confirmed that demand elasticity for both commodities differed between compensated and uncompensated budget conditions, also when the number of choices considered was matched, and showed that these elasticity differences emerge early in the sessions. These differences in demand elasticity were driven by a higher choice rate and an increased reselection bias for the preferred commodity in compensated compared to uncompensated budget conditions, suggesting a budget context effect on relative valuation.

## Introduction

How do we choose between different goods out of the vast number of options available? Imagine a shopping trip to the supermarket where one is faced with the hard task of selecting a combination of goods that falls within a pre-specified budget. Though one might like to spend the whole budget on ice cream (the item that would yield the highest increase in subjective value, or, in economic terms, *utility*), such a decision, besides being quite unhealthy, would ignore that even though one likes ice cream the most, one likes other items as well. At some point, spending money on even more ice cream is not going to increase one’s subjective happiness as much (decreasing marginal utility [1,2,3]) as spending it on different items. Thus, in order to maximize overall utility, one would be best off splitting the available budget according to one’s relative preference for the different items.

Besides relative preferences, other factors also influence budget allocation. Classic demand theory states that the allocation of choices amongst two commodities should be influenced firstly by the relative price of those goods: the more expensive a good is, the less it is purchased. To describe how sensitive a consumer is to price changes in a commodity, the demand elasticity of such a good can be calculated from the number of purchases of this commodity made at different prices. Demand elasticity can thus be understood as how flexible a consumer is about purchasing a specific commodity. Low elasticity—a small change in purchases with rising or falling prices, is typically found for commodities that a consumer absolutely needs, such as staple food items or transportation. The converse, high elasticity, applies to those commodities for which consumers exhibit strong changes in purchases with fluctuating prices such as for seasonal or luxury food items.

However, as stated in the example above, consumers usually do not consider purchases of only one commodity, but rather select a distribution of purchases amongst several alternatives on offer. In a situation where a consumer is choosing between commodities A and B, e.g. apples and oranges, the demand elasticity of apples and oranges can be evaluated concurrently by changing the price of just apples, just oranges or both commodities at the same time. In economics terms, the effect of a price change of one of the commodities on offer is twofold: firstly, the relative price of the commodities changes, which is predicted to lead to substitution effects in bundle composition (here, a bundle is a set of apples and oranges). Because apples can be substituted by oranges, a decision maker may compensate the price increase of the now relatively more expensive commodity by increasing consumption of the now relatively cheaper commodity. Secondly, the budget from which the consumer makes purchases, also limits the choices she can make. Imagine that the price of apples goes up, while the price of oranges goes down. This means a consumer can now afford less apples, but more oranges. She could compensate the reduced purchasing power for apples by adding more oranges to the bundle. Assuming for the moment that our consumer prefers apples to oranges, e.g. chooses more apples than oranges when the price of both commodities is equal (revealed preferences; for example 30 apples and 10 oranges at €4 each from a budget of €160). If prices change and apples become more expensive (€5 per apple) and oranges become less expensive (€3 per orange) at the same time, this might affect her relative preference for the commodities. She might want to purchase some more oranges, and fewer apples, for example 25 apples and 15 oranges (substitution effect). However, it would not be possible to purchase the original nor this altered bundle from the same budget of €160 again (purchasing the original bundle of 30 apples and 10 oranges would now cost €180 while purchasing the altered bundle would cost €170; both bundles thus exceed the budget). As such, an uncompensated price change gives rise to an income effect on choice allocation in addition to a substitution effect: in our example, the budget (income) limits the choices that can be made and forces a second change in relative consumption over and above a change that might have been made due to altered prices. To offset budget effects on choice allocation and isolate the substitution effect, the available income can be adjusted. In the above example, increasing the budget by €20 so that the adjusted budget would now be €180 would allow the consumer to purchase the same bundle of 30 apples and 10 oranges. An everyday example of a price change with budget compensation is the salary adjustment to compensate for inflation rate that allows employees to conserve their purchasing power. Hence, compensated price changes present a shift in commodity prices that is accompanied by a concurrent adjustment of the budget upward or downward so that the originally chosen bundle could in principle be reselected within the new budget constraints, isolating the substitution effect from the income effect. Typically, when budgets are adjusted to allow reselection of the previously chosen bundle under the new price regime, different choice distributions and thus demand elasticities are observed compared to conditions where prices are changed without compensating budgets, in line with a substitution effect that persists even when the income effect is cancelled by budget compensation [4,5].

However, it is unclear precisely how and when budget compensation affects consumers’ choices between commodities over and above an influence of price changes. In many empirical tests of budget effects with animal consumers, subjects make sequential choices between commodities [4–7]. That is, they select one of two (or more) commodities in each choice opportunity, and repeat choices until their budget expires or another stopping criterion is met. The final obtained bundle is equal to the total number of selected items from each commodity across the sequence of choice opportunities. Thus, in such studies, as in typical studies of human individual demand [8], elasticity is calculated over the total of sequential choices made from a budget, potentially neglecting differences in sequential choice structure between uncompensated and compensated price change conditions. More precisely, for sequential choices, as compensating budgets changes the number of attainable commodities relative to uncompensated conditions (if the budget is increased, the total number of choices is increased, too), it remained unclear whether budget compensation has an effect on demand elasticity by 1) the trivial effect of sampling from a different number of choices for compensated versus uncompensated budget contexts or by 2) actually changing the sequential choice structure, putatively reflecting altered relative valuation of the commodities on offer. We elaborate on these two explanations, and their different predictions for calculations of demand elasticity over different sets of trials, in the following:

1) to illustrate the possibility that income-effects on price elasticity estimates are merely the consequence of sampling from different choices, consider the following example (adapted from above): a consumer has a budget/income of €160 and buys 30 apples and 10 oranges, each costing €4. Now, prices change and apples cost €5, oranges €3. In an income-uncompensated condition, this consumer still has €160 to spend, and now buys, say, 20 apples and 20 oranges. In an income-compensated condition, the budget is adjusted to €180, so that the consumer has €20 more to spend relative to her original income. Assume that the consumer now buys 24 apples and 20 oranges. Comparing the elasticity measures between the income-compensated and—uncompensated conditions would yield different elasticity estimates for apples, suggesting that income-effects affect choice distributions over and above substitution effects. Now suppose that the consumer has spent the first €160 in the income-compensated condition on 20 apples and 20 oranges, and spends the remaining €20 on apples only. If this was the case, calculating price-elasticity across the budget shared between both conditions (€160) would yield exactly the same elasticity estimates in both conditions; the different elasticity estimates obtained when considering the entire choice set would merely be the result of the final €20 spent on apples. In other words, the difference in elasticity estimates when considering the entire choice set would simply be the consequence of the extra set of choices available after budget extensions.

This possibility implies that choice allocation, and changes in choice allocation due to price changes, should be identical in compensated and uncompensated budget conditions when considering *only* those choices that are shared by both conditions. Thus, this theory predicts that, if only the shared choices would be considered, no difference in elasticity estimates would be expected.

2) Alternatively, it is possible that the size of the budget immediately affects the consumer’s sequential choice structure. This could be the case if the budget context affects the relative valuation of the choice options. If consumers indeed have an internal representation of the current budget/price set and if this representation affects relative valuation of the commodities on offer, it is to be expected that this results in differences in choice allocation that are visible throughout the session and thus result in different elasticity measures (for compensated vs. uncompensated budget conditions) evaluated over choice sets of *any* reasonable size. Such differences in demand elasticity estimates in this case do reflect a true income effect on choice allocation between commodities. To illustrate this possibility, take again the above example: how do choices change in response to price alterations for apples (€4→€5) and oranges (€4→€3) in uncompensated (budget €160→€160) and compensated (budget €160→€180) income conditions? If relative valuation of apples and oranges depends not only on their price, but also the available budget, a difference in budget under similar price structures should thus change choice allocation. In other words, an apple for €5 may have a different relative utility for a consumer if she has a budget of €160 compared to a budget of €180. If, say, apples for €5 are valued higher when the consumer has €180 to spend (compensated income condition) than when she has €160 to spend (non-compensated income condition), she would purchase more apples in the compensated condition. Importantly, once the consumer has learned the price and budget condition she is in, she would buy relatively more apples in the compensated than uncompensated income condition from the beginning of a choice sequence on. Thus, when comparing the choice allocations within the first €160—the budget common to both conditions, our example consumer would purchase relatively more apples in the compensated than the uncompensated income condition. The income-effect on price-elasticity should therefore be detectable in any subset of choices, and should become evident long before the budget runs out.

To disambiguate between these two predictions, it is therefore imperative to investigate the evolution of choice distributions across a fixed range of consecutive choices, and to use comparable choice sets when computing demand elasticities in order to isolate budget effects on choice distributions from other temporal dynamics.

We approached the question whether budget adjustment (compensation) independently affects the temporal dynamics of choice distributions by training rats on a classic demand task. In this task, animal consumers spend a budget of nosepokes between two commodities, chocolate- and vanilla-flavoured soymilk [4,5], in sequential blocks (N = 7 blocks in total) of 10 daily sessions. As rats cannot be explicitly instructed about the budget but have to learn the current extent of the budget by experiencing it, budgets and commodity prices were kept stable for 10 daily sessions in such a block but changed between blocks (see below). We used rats to study economic choices because, as we and others have pointed out before [9,10], animals are a “means to probe the elementary principles of microeconomic theory: if these basic principles fail to account for the behaviour of simple organisms, such as rats or pigeons, in simple choice situations, such as Skinner box experiments, how can they be trusted in much more complex situations involving much more complex organisms, such as our worldwide economic systems with human actors?” (p. 6, [9]). Thus, a successful application of demand theory to rat behaviour would lend further support to the versatility of economic theory, fostering its usefulness as a tool to formalize animal behaviour.

As the chocolate-flavoured soymilk contained more sugar than vanilla-flavoured soymilk, we expected rats to show a (baseline) preference for chocolate, reflected in choice proportions above chance level when the prices for both commodities were equal. Consequently, we expected less elasticity in demand in the face of price changes for the preferred, chocolate-flavoured soymilk compared to the vanilla-flavoured soymilk. Given that rats had access to food and water outside the experiment, we furthermore expected that the commodities would be treated as non-essential and substitute for each other, thereby predicting positive cross-price elasticity, e.g. an increase in consumption of commodity A when the price of commodity B is increased and vice versa. However, considering that animals have a tendency to explore and alternate in an option space, we predicted that substitution would be imperfect, e.g. that animals would not solely choose one option in any budget/price condition. Based on previous work [4,5], we expected to find reduced (i.e. less negative) demand elasticity for the preferred commodity in compensated compared to uncompensated price changes, because budget compensation cancels the income effect on bundle selection, thus putatively reducing pressure to alter consumption in the face of changed prices. In terms of the budget effect on choice structure, we favor alternative #2 as outlined above and thus expected that time-resolved choice ratios under compensated budgets would differ from those obtained with uncompensated budgets when assessed over matching choice sets. This implies that budget effects on demand elasticity should also become apparent in the comparison of these matched choice sets and that they are not merely the result of enhanced (or reduced) opportunity for commodity selection in extended (or shortened) budgets but instead reflect a real influence of budget context on relative commodity valuations, resulting in altered choice patterns between these contexts.

## Materials & Methods

### Subjects

A group of 8 male Long-Evans rats (Janvier Labs, St. Berthevin, France) was used in these experiments. Rats were housed in groups of four animals per cage maintained at 22°C on an inverted 12h:12h light/dark cycle (lights off at 07:00 AM). All rats weighed 240 to 260 g at the beginning of the study and were food restricted after the first four weeks to 15 g per animal per day (delivered as a 60g allotment per cage ca. 1 hr after the experiment finished). Weights were monitored during the whole experiment to ensure continued weight gain. Water was available ad libitum in the home cage at all time.

### Experimental setup

**Fig 1:**
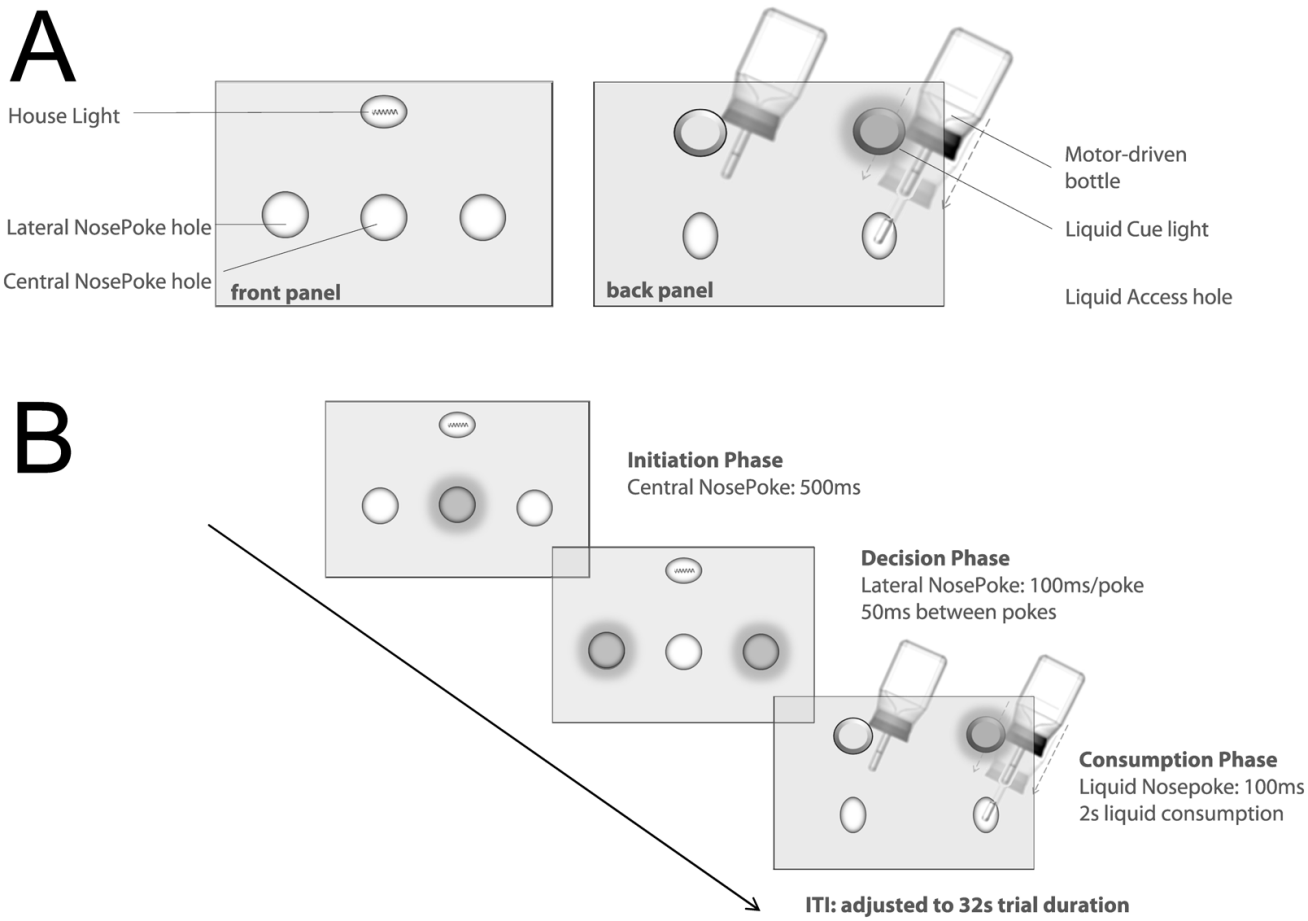
Schematic illustration of operant cage layout and task outline A) Front panel and back panel of the operant chamber are shown. Front panel consisted of three nosepoke holes (one central, two lateral) each containing a light-emitting diode (LED) as an operant conditioning cue. The back panel on the opposite side of the operant cage was fitted with two laterally placed liquid access holes, each allowing a motor driven bottle to be lowered into the hole to provide time-restricted access to the selected liquid. B) One trial consisted of 3 phases (initiation phase, decision phase and consumption phase) starting with the illumination of the LED in the central nosepoke hole. After completion of phase three, a flexibly adjusted inter-trial-interval was imposed so that each trial lasted exactly 32 seconds (see main text for further information).

Experiments were conducted in operant chambers (28 x 23 x 23 cm; Med Asssociates Inc., Georgia, VM, USA) under red light conditions. Each chamber was equipped with three nosepoke units (i.e. holes containing photobeams able to detect and signal when the rat enters the hole with its snout). These nosepoke units were horizontally arranged on one side of the chamber (Front panel; see Fig 1A), containing a yellow light-emitting diode (LED) at the rear of the hole used as visual cue. The opposite side of the chamber (Back panel) was equipped with two liquid access holes, each providing experimenter-controlled access to a liquid dispenser bottle and likewise fitted with an infrared photobeam for head entry detection. Motorized drivers could lower these bottles individually into the liquid access holes to make the liquids temporarily available to the animals. Liquids consisted of soymilk (Alpro, Düsseldorf), either vanilla-flavoured (55kcal/100ml, diluted to a ratio of 1:3 in water, final concentration: 13,8kcal/100ml), or chocolate flavoured (63kcal/100ml, diluted to a ratio 2:3 in water, final concentration: 25,2kcal/100ml). These different dilutions were chosen to facilitate the establishment of choice biases based on taste and/or sugar concentration. A cue light was located above each liquid access hole, indicating bottle availability when lit. In addition, the chamber was illuminated with a 15-W light bulb serving as a house light, which was turned off when the experiment started and turned on again at the end of the session. All inputs, outputs and events were time-stamped, recorded by the software MedIV-PC (Med Asssociates Inc., Georgia, VM, USA) and stored for offline analysis.

### Task structure & shaping

Every rat was trained for one session per day on weekdays. Experimental sessions were conducted in the dark part of the day/night cycle; animals were transported in a light-shielded cart and handled strictly under dim red light conditions. After two weeks of habituation, animals were trained to nosepoke and collect liquids on a fixed-ratio reinforcement schedule (FR1-FR4, ca. 30 min per day) in seven training steps with increasing complexity (see S1 Table) leading up to the following task structure (Fig 1B):

#### Initiation phase

After initiating a trial by making a nosepoke with a duration of at least 500ms in the central nosepoke unit, the two lateral nosepoke holes became active, indicated by the illumination of the corresponding cue LEDs in the nosepoke holes.

#### Choice phase

After meeting the respective FR requirement set by the current price level (a series of 3–5 nosepokes), in one of the lateral nosepoke holes (duration >100ms), separated by 50 ms delays during which the LED in the nosepoke holes turned off briefly, the liquid availability was signalled by a cue light on the opposite site turning on.

#### Consumption phase

A nosepoke of 100ms in the corresponding liquid access port led to the delivery of the bottle containing a either a vanilla-flavoured soymilk or chocolate-flavoured soymilk solution, depending on which side was chosen. Bottle access was available for 2s. Afterwards, the bottle was retracted out of reach of the animals.

#### Inter-Trial interval

Following bottle retraction, an ITI was implemented before the next trial started, during which interaction with the operant elements had no programmed consequences. Each trial lasted exactly 32 seconds.

In the final task structure, the number of nosepokes required in the choice phase was used as a proxy for price, or cost, and varied between liquids (commodities) and task conditions (price regime; see budget and price conditions below for details, Table 1). For example, if one commodity was priced at three nosepokes, the animal had to make three consecutive nosepokes of 100ms each in the lateral nosepoke hole. We define a choice as the selection of one liquid after having paid the price for obtaining access to that bottle. Animals had a fixed budget per session that could, however, vary across sessions (see budget and price conditions for details). The budget corresponded to the number of nosepokes available during a session. For example, if a rat had a budget of 160 nosepokes in a session and used three nosepokes to obtain liquid access in the first trial, it was left with 160–3 = 157 nosepokes for the remaining trials. Each experimental session started with 8 forced-choice trials (4 on each side; only one lateral nosepoke unit was illuminated and programmed to lower the corresponding bottle, poking in the unlit lateral nosepoke unit reset the trial) to ensure that the rats received equal exposure to both outcomes and associated ratio requirements, followed by a variable number of free-choice trials (both lateral nosepoke units active). Forced-choice trials were selected pseudorandomly, e.g. they were selected without replacement from a list of four choices, consisting of two chocolate and two vanilla forced choices. This 4-trial block was repeated twice (allowing for another random, different order in the second block). The first trial thus was always chosen randomly and the same choice could be presented on no more than 4 consecutive trials. If a forced-choice trial resulted in a failed trial, it was repeated without changing any parameters. After completing the forced-choice trials, in the free-choice trials the animals could choose between responding at both lateral nosepoke units to acquire access to the corresponding liquid bottles. An experimental session was terminated when the entire budget was spent, or else after 60min. Session were included for analysis when at least 85% of the budget was used.

**Table 1:** Ordering of price ratio regimens per rat and number of included sessions.

Phase	I	II	III	IV	V	VI	VII
Animals; #Sessions	31	47	48	44	48	42	48
#1 #8; 47/52	4:4; 2/5	5:3; 7/8	3:5; 8/8	4:4; 7/8	5:3C; 8/8	3:5C; 7/7	4:4; 8/8
#3 #5; 0/48	4:4; 0/4	5:3C; 0/8	3:5C; 0/8	4:4; 0/6	5:3; 0/8	3:5; 0/6	4:4; 0/8
#2 #7; 54/52	4:4; 7/6	3:5; 8/8	5:3; 8/8	4:4; 8/7	3:5C; 7/8	5:3C; 8/7	4:4; 8/8
#4 #6; 54/0	4:4; 7/0	3:5C; 8/0	5:3C; 8/0	4:4; 8/0	3:5; 8/0	5:3; 7/0	4:4; 8/0

X:Y: price in nosepokes for chocolate/vanilla. C: compensated budget conditions. Stricken rat identifiers signify rats that were excluded from the final analyses. Sessions were included when >85% of the budget was spent. Numbers in each second line signify included sessions per block (max = 8); in first column: total included sessions (max = 56)

### Budget and price conditions

The experiment consisted of seven blocks of 10 consecutive daily sessions (hereafter referred to as phases), with different price regimes and different budgets (compensated, non-compensated) adding up to a total of 70 testing days. The default price (‘baseline’ phases) for both commodities was 4 nosepokes (FR, 4:4, chocolate:vanilla), with a budget of N = 160 nosepokes. Consequently, rats could obtain access to vanilla and chocolate in any combination up to 40 times daily. All animals started off in a baseline phase, followed by two experimental phases (one phase with a price increase for one commodity and concurrent price decrease for the other [FR 5:3], followed by another phase with inverted prices [FR 3:5], order counterbalanced between rats). The price changes in these two phases were either compensated, for half of the animals, or uncompensated for the other half (see Table 1). The fourth phase was again a baseline phase, after which the two experimental phases were repeated, but now without/with budget compensation, respectively. The final, seventh phase was again a baseline phase, resulting in a fully counterbalanced test design with 2 rats per cell.

In the uncompensated budget conditions, rats retained the original budget of 160 nosepokes available in the baseline conditions. In the compensated budget conditions, each animal’s budget was adjusted individually so that the amount of each commodity chosen by the animal during the preceding baseline phase could theoretically be purchased again under the new price regime. For instance, imagine a rat spending 120/160 nosepokes on chocolate, and 40/160 nose-pokes on vanilla in the baseline phase, thus purchasing 30 chocolate and 10 vanilla bottle drops (each bottle drop costing 4 nosepokes). In order to allow the rat to obtain, again, a bundle of 30 chocolate and 10 vanilla bottle drops in the compensated 5:3C condition, the budget would need to be adjusted to 180 nosepokes (150 nosepokes would now yield 30 chocolate bottle drops, 30 nosepokes would now yield 10 vanilla bottle drops). This example illustrates a budget extension (from 160 nose-pokes in the baseline condition to 180 nose-pokes in the compensated budget condition), but budget contractions (reduction of budget) occurred as well, namely in the 3:5C condition (using the example above, the budget would be reduced to 30*3 + 10*5 = 140 nosepokes). From each experimental phase, the first two days were discarded to allow the rats to adapt to the new price- and budget-regime (see results for

### Data analysis

For elasticity calculations, the absolute number of choices for each commodity was averaged across sessions within a phase per rat. For choice proportion calculations, proportions were calculated per rat per session and subsequently averaged across sessions within a phase per rat. In order to guard against outliers in the data, we estimated the mean number of choices for chocolate by running a bootstrap analysis. For N = 5000 repetitions, we randomly drew 8 sessions with replacement and calculated the mean. Of the resulting normal distribution of means, the median value was entered into the subsequent demand elasticity calculations.

Own-price elasticity was calculated using linear regression on the log-transformed number-of-choices/price-ratio pairs as given by the formula:

$$\text{log}\;q_{i}\;=\text{log}\;\alpha \;+\;\varepsilon \;\text{log}\;(p_{i}/p_{j})$$ (1)

with q_i_ as the quantity of chocolate chosen, p_i_/p_j_ as the price ratio for chocolate over vanilla per price/budget regime, and α as a constant [4]. Price elasticity values ε<-1 indicate elastic demand, and ε>-1 indicate inelastic demand. Cross-price elasticity employs the same formula, but substitutes q_j_ (choices for the other commodity) for q_i_ as the target variable.

To estimate the dynamic development of choice ratios with increasing trial number, we calculated running averages of the choice proportion for chocolate by dividing the number of trials on which chocolate was chosen in the past 10 trials by 10, and sliding this analysis window along the set of trials. To compare between conditions, we pooled all running average choice proportion timeseries for all sessions for rats in a condition (maximally 8*8 sessions) and ran another bootstrap analysis on the data, this time drawing N = 50 sessions, with replacement, per bootstrap iteration and taking the average. We report the median of the resulting bootstrap distributions, and their 95% confidence intervals (adjusted by Bonferroni correction for the number of time points examined) by plotting the lower and upper percentile corresponding to the adjusted two-tailed p-levels of 0.05 in the sorted bootstrap populations. Significance is tested by dividing the difference in bootstrapped means between conditions by the weighted standard deviation and evaluating the result, per time point, as |Z| > 1.96 (uncorrected). Significant differences are flagged if the Z-level exceeded 1.96 after Bonferroni-correction (in our case, |Z|>3.07).

### Ethics statement

All animal procedures adhered to German Welfare Act and were approved by the LANUV (Landesamt fuer Natur-, Umwelt- und Verbaucherschutz, North Rhine-Westphalia, Germany, Case number 84–02.05.30.12.064).

## Results

### Basic choice distributions

To allow the rats to experience the new, sometimes adjusted budgets for each experimental block of sessions (phase), we excluded the first 2 sessions from each phase from the analysis. Indeed, the coefficient of variation across subsequent sessions, averaged across phases and rats, almost halved from session 1–2 to session 3–4 and remained lower afterwards. A paired-sample t-test comparing the coefficient of variation (CV) of sessions 1–2 against the average of the four subsequent 2-session blocks turned out significant: (*t*_(17)_ = 2.34; p = 0.03, S1 Fig, only complete data [i.e. all sessions included for a given rat/phase combination] were considered). Consequently, commodity choices were extracted from the third through tenth session per price regime only and subsequently averaged, for each phase, across rats and sessions (Table 2). Due to a high number of incomplete sessions, two rats were permanently excluded from analysis. The subsequent analyses are thus based on N = 6 rats. Only sessions in which >85% of the budget was spent were included, for a total of N = 306 out of a total of 336 sessions (6 rats, 8 sessions, 7 phases). The session with incompletely spent budgets occurred in all phases, not just the sessions with extended budgets. We found that the number of nosepokes made was significantly higher in the condition with extended budgets (5:3C; 184±1.1) compared to the sessions with similar prices but without budget compensation (5:3; 159±0.9; *t*_(93)_ = -20.1, p < 10^–30^), suggesting that rats indeed experienced budget extensions.

**Table 2:** Proportional choice for chocolate and budget (un)spent per phase.

Phase	B1	B2	B3	5:3	5:3C	3:5C	3:5
#Sessions	31	44	48	46	47	45	46
% Choc Choices	76.0 ±7.3	86.2 ±2.2	87.3 ±2.2	61.2 ±7.1	69.5 ±4.6	90.2 ±1.6	90.6 ±1.9
Budget Spent	162 ±1.8	159 ±0.4	159 ±0.3	159 ±0.9*	184 ±1.1*	137 ±1.0	159 ±0.5
Budget Unspent	-1.73 ±1.8	1.02 ±0.4	0.58 ±0.3	0.93 ±0.9	0.04 ±0.5	-0.35 ±0.9	-0.75 ±0.5

Numbers indicate mean ± standard error of the mean, sem.

B: baseline; X:Y: price for chocolate/vanilla. C: compensated budget conditions.

*: significantly different, t_(93)_ = -20.1, p < 10^–30^

^¶^: significantly different, t_(84)_ = -17.4, p < 10^–20^

### Price changes influence choice distributions

**Fig 2:**
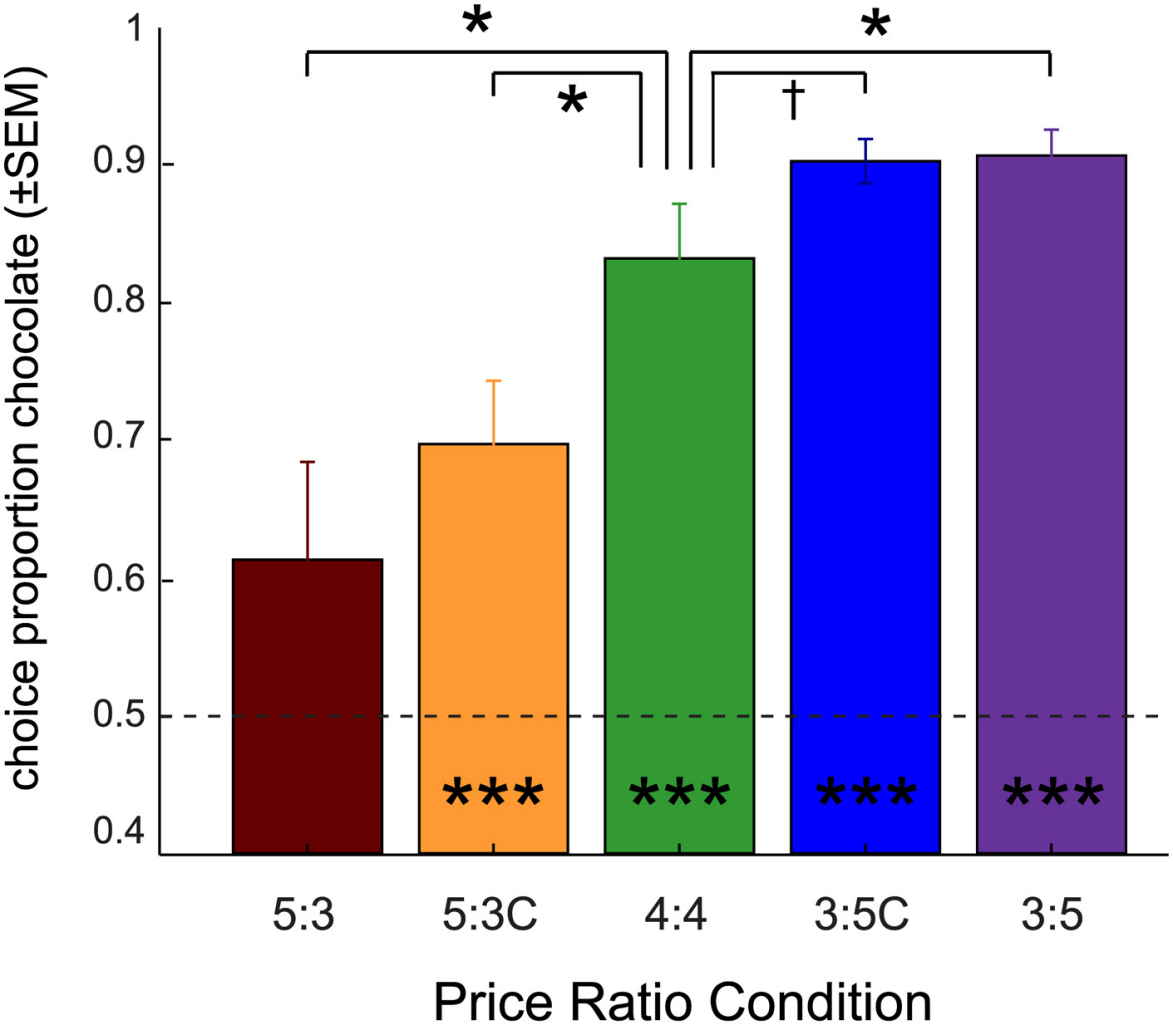
Choice proportion for chocolate per experimental condition Group average preference for the chocolate solution expressed as the proportion of the budget spent (±SEM) on obtaining chocolate, per rat averaged over sessions (N = 8 sessions per condition). In green, the three baseline conditions (4:4 price ratio) were averaged into a single measure per rat. *: significantly different from baseline; p<0.05 paired-sample t-test. †: significantly larger than baseline; p<0.05 paired-sample t-test, one-sided. Dashed line: indifference point, equalling a choice proportion of 0.5. ***: significantly different from chance, p<0.001 one-sample t-test vs. 0.5.

Across the seven price/budget regimes, when analysed at the group level, rats preferred chocolate at choice ratios significantly above chance (one-sample t-test against 0.5, all p<.01), in all phases except for the 5:3 condition (Fig 2). Compared to chocolate choices averaged across the three baseline periods, choice proportions for chocolate in the 5:3 and 5:3C conditions were significantly lower (all p<0.05, paired-sample t-test), indicating that demand for chocolate decreased with increasing price. In the 3:5C condition, the choice proportion for chocolate was only slightly increased relative to baseline (see Table 2, *t*_(5)_ = 2.14; p<0.05, one-sided), while it was significantly higher compared to baseline in the 3:5 condition (*t*_(5)_ = 3.14; p<0.05). These results confirm that price changes influence choice distributions, even in the budget condition where the original bundle could have been purchased (cf. [4,5]).

### Price elasticity differs between uncompensated and compensated conditions

**Fig 3:**
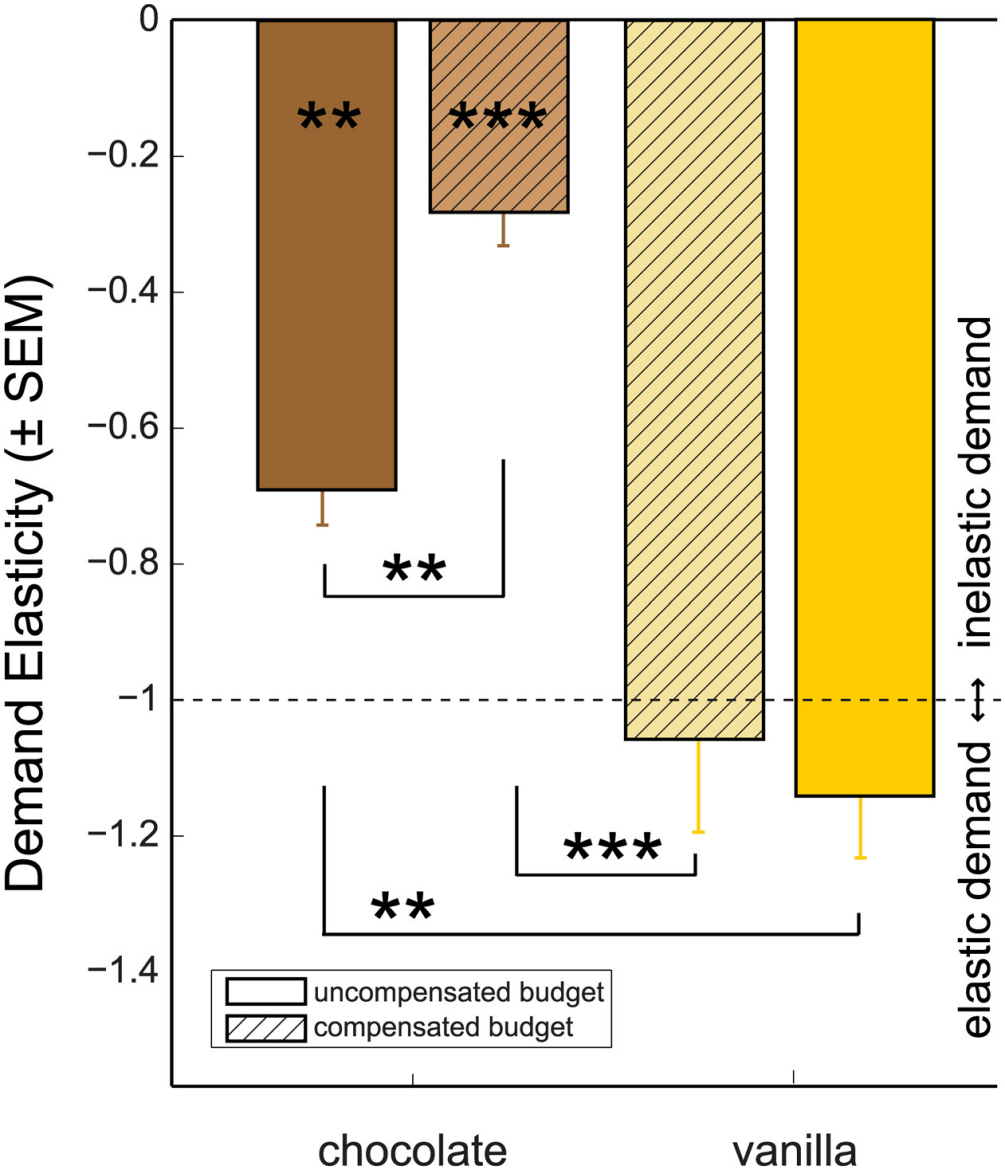
Demand elasticity (ε) for chocolate and vanilla solutions Group averaged elasticity estimates (±SEM) calculated per rat using averaged choice data across all sessions, separated per condition. Demand elasticity for chocolate (brown) was significantly less elastic than unit elasticity (ε = -1) under uncompensated (open bars; **: p<0.01, one-sample t-test vs. -1) and compensated budgets (hatched bars, ***: p<0.001). Demand was significantly less elastic for compensated vs. uncompensated budget conditions (**: p<0.01, paired-sample t-test). For vanilla (yellow), demand was significantly more elastic than for chocolate, both in the uncompensated (**: p<0.01, paired-sample t-test) and uncompensated budget condition (***: p<0.001).

**Fig 4:**
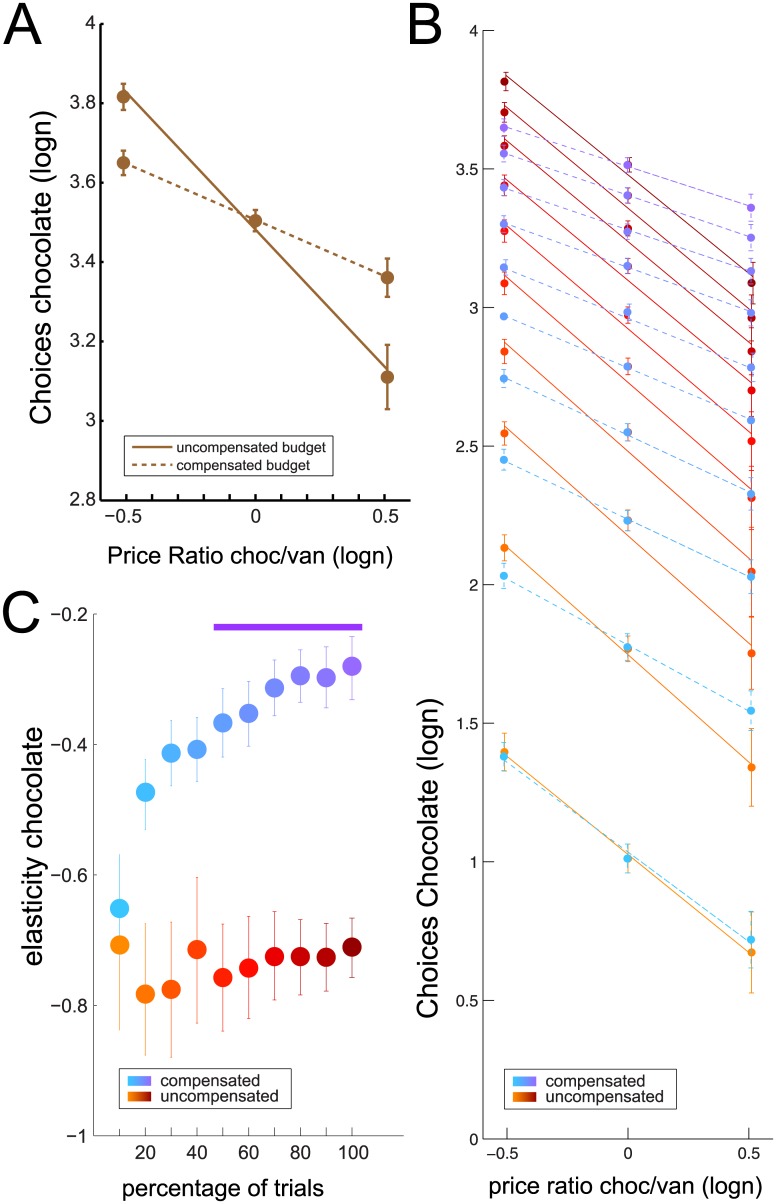
Demand elasticity for chocolate over entire and cumulatively spent budgets A) Scatterplot of log-transformed choices for chocolate (mean ± SEM across rats) by log-transformed price-ratios (chocolate/vanilla) for uncompensated (solid line) and compensated budget conditions (dashed line). Please refer to Fig 3 for significance of the differences. B) as in A), but now displaying log-transformed number of chocolate choices calculated over 10 cumulative steps of 10% of the entire budget. Colour coding indicates percentage of budget considered: from yellow to brown (solid lines): 10–100% of budget in the 5:3 condition; from blue to purple (dashed lines): 10–100% of budget in the 5:3C condition. C) Elasticity estimates for the chocolate choice data shown in B) with cumulative uncompensated budget fractions in hot colors, and cumulative compensated budget fractions in cool colors. Error bars: SEM. Purple bar: significant difference incremental paired sample t-test (1^st^ time point: p<0.05, 2^nd^ time point p<(0.05/2), 3^rd^ time point p<0.05/3 etc.; see text for details).

Using the distributions for chocolate and vanilla choices, averaged per condition per rat, we calculated price elasticity (ε) for chocolate (the preferred commodity) and vanilla. As mentioned, price elasticity values ε<-1 indicate elastic demand, and 0>ε>-1 indicate inelastic demand. Price elasticity for chocolate with budgets uncompensated (ε_choc/uncomp_) was -0.69 ± 0.05 (mean ± sem, all individual regression coefficients significant at the p<.05 level, Figs 3 and 4A). Price elasticity in the uncompensated budget condition was significantly different from price elasticity under compensated budgets (ε_choc/comp_; -0.28 ± 0.05; *t*_(5)_ = -5.31; p<.005, paired-sample t-test). Both under uncompensated and compensated budget conditions, demand for chocolate was inelastic (*t*_(5)_ = 5.93; p < .005 and *t*(5) = 14.8 p < .001, respectively, for a one-sample t-test against -1). In contrast, demand for vanilla was significantly more elastic than demand for chocolate in both uncompensated (ε_van/uncomp_: -1.13 ± 0.09, *t*_(5)_ = -5.80; p < .005 against ε_choc/uncomp_, paired-sample t-test) and compensated budget conditions (ε_van/comp_: -1.06 ± 0.13, *t*_(5)_ = -7.55; p < .001 against ε_choc/comp_), though elasticity at the group level neither differed significantly from unit elasticity (*t*_(5)_ = -1.56 and -0.42; n.s.) nor differed between uncompensated and compensated budget conditions (*t*_(5)_ = -0.43; n.s.).

Cross-price elasticity was 0.72 ± 0.05 for chocolate and 1.11 ± 0.07 for vanilla under uncompensated conditions (all mean ± sem, S2 Fig). For compensated budget conditions, the cross-price elasticity was 0.30 ± 0.05 for chocolate and 1.03 ± 0.12 for vanilla, confirming that both goods can be considered substitutes, and that vanilla substitutes more for chocolate than vice versa under both uncompensated (*t*_(5)_ = -5.80; p < .005) and compensated (*t*_(5)_ = -7.55; p < .001) budget conditions.

Thus, price elasticity differed between the uncompensated and compensated budget conditions. However, importantly, as mentioned in the introduction, it is unclear what produced these differences in price elasticities. Alternative #1 (cf. introduction) holds that considering the entire choice set for compensated and uncompensated budget conditions might produce differences in demand elasticity that can be explained just by the act of comparing choice sets with unequal number of choices. In contrast, it is also possible that the budget context had an independent effect on commodity selection, perhaps through directly affecting relative valuation of the commodities on offer (alternative #2). The crucial question thus is whether the relative distribution of choices between chocolate and vanilla, presumably reflecting relative valuation, differs between compensated and uncompensated budget conditions when examining choice sets that are matched for price and number of choices. If differences in choice patterns between compensated and uncompensated conditions, and in the subsequent estimates of demand elasticity, emerge early in a session, it would support the hypothesis that the budget context has an effect in of itself on the relative valuation of the commodities, so that rats value chocolate and/or vanilla differently in the compensated than in the uncompensated condition and adjust their choices accordingly.

To determine whether the effect of budget adjustment on price elasticity was merely the consequence of the difference in the attainable number of choices, or whether it really reflected differences in sequential choice structure, we computed elasticity across a broad range of choice sets of different size containing cumulative choice distributions in steps of 10% of trials within a session (Fig 4B). To assess the difference between the cumulative elasticity estimates for both budget conditions (Fig 4C) statistically, we ran a repeated-measures ANOVA with budget condition and incremental 10% step as repeated measures. We found a significant main effect for budget condition F_(1,45)_ = 12.2, p = 0.017, partial η^2^ = 0.71, a significant main effect for trial step F_(9,45)_ = 5.68, p < 0.001, partial η^2^ = 0.53 and a significant interaction F_(9,45)_ = 3.57, p = 0.002, partial η^2^ = 0.41. Some caution is warranted, as the cumulative nature of the data makes the incremental trial steps highly correlated. To isolate which region of the data was significantly different, we employed a paired-sample t-test with incremental alpha level correction, setting α at 0.05/1 to detect the first significant divergence between the budget conditions (found here at 50% of trials). Subsequent data points (60%-100%) were then tested for significance with a corrected α level of 0.05/2 for the second, 0.05/3 for the third etc. resulting in a corrected α level of 0.05/6 = 0.0083 for the 10^th^ data point, which was passed (t_(5)_ = 5.79, p = 0.0022). We thus found that elasticity of demand for chocolate quickly diverged between the compensated and uncompensated conditions and remained at significantly different levels throughout the rest of the session (Fig 4C), suggesting that budget effects on choice allocation, independent from the effects of relative commodity prices, became evident already early during sessions, and were not necessarily the consequence of the difference in session length and/or numbers of choices. However, these blocks of 10% of trials consisted of unequal numbers of trials, as rats performed different numbers of trials in the compensated and uncompensated price change sessions compared to the default of 40 choices for a 4:4 baseline session with a budget of 160 nosepokes.

**Fig 5:**
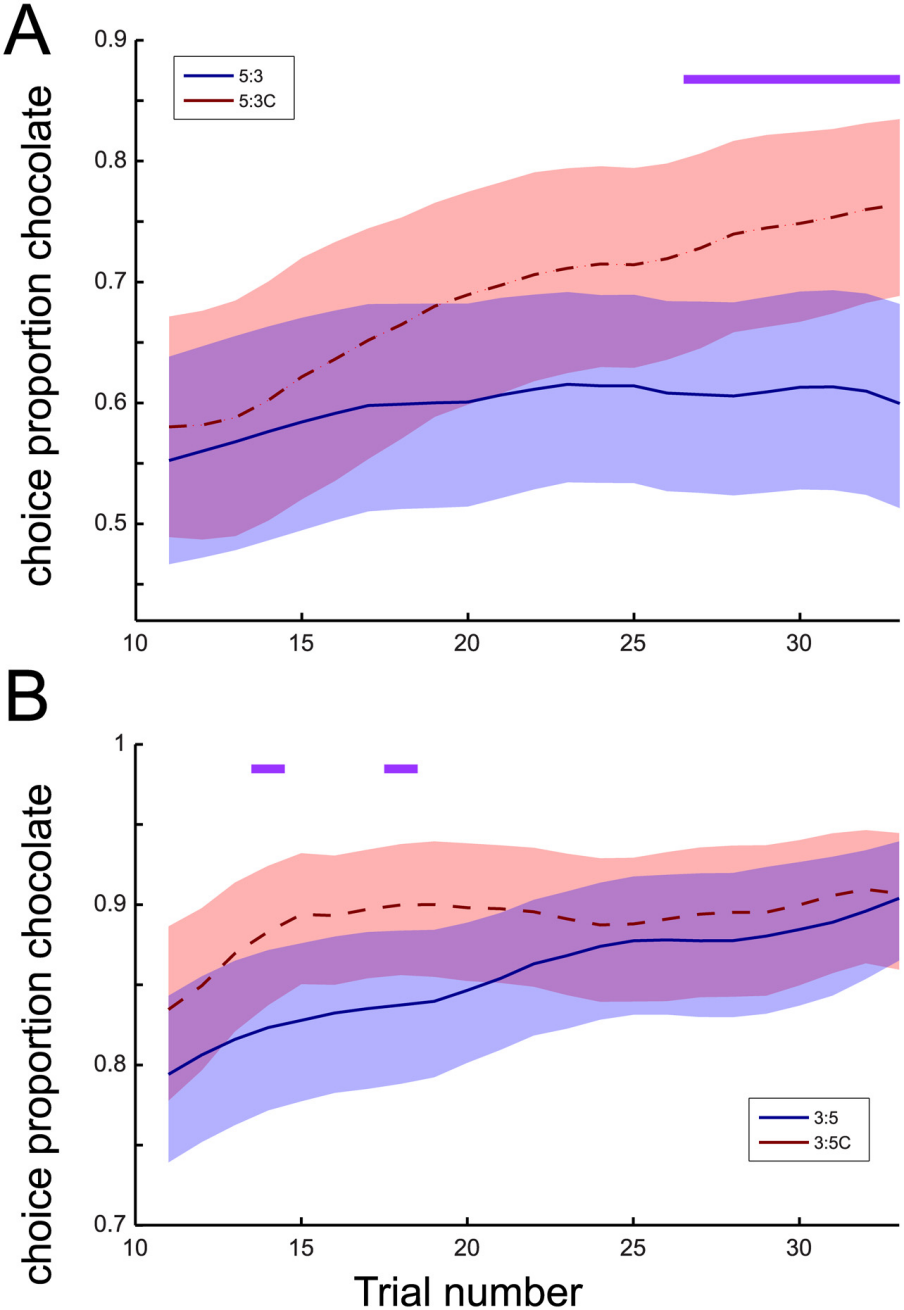
Time-resolved proportion of choice for chocolate as a function of trial number A) Time-resolved proportion of chocolate choice was modelled from trial 11 to 35, integrated over choices in the last 10 preceding trials. Mean choice proportion estimates with bootstrapped 95% confidence intervals for the conditions with expensive chocolate are displayed for the 5:3 (uncompensated, blue line) and 5:3C (compensated, red dashed line) conditions. Purple bar: Difference in Z-value > 1.96 (p<0.05, Bonferroni-corrected for multiple comparisons to |Z|>3.07). B) as in A), but now for choice proportions estimated for the conditions with inexpensive chocolate (3:5, uncompensated, blue line; 3:5C, compensated, red dashed line).

In order to show the underlying dynamics in relative valuation over the course of a behavioural session on a trial-by-trial level, we computed a moving average index of choice proportions for chocolate, computed over a sliding block of 10 trials, comparing conditions only up to trial 33. This number of choices was reached in >95% of sessions and thus can be safely used to compare between all conditions without running the risk of comparing unequal sets of trials (sessions included in the comparison) between conditions. Using bootstrap resampling techniques (cf. [11]), 95% confidence intervals on the choice time series over trials were estimated. Fig 5A shows that, from trial 28 onwards, time-resolved choice proportions for chocolate were significantly higher in the 5:3C than the 5:3 condition (|Z|>3.07; p<.05, Bonferroni corrected for multiple comparisons), confirming a budget effect on choice distributions across comparable trial sets. A similar sliding window analysis was performed for choice proportions against budget spent, employing a budget integration window of 30 nosepokes (S3A Fig), with comparable results. A reflection of this result was found when the analysis was repeated for the 3:5 vs. 3:5C condition. Now, with relative inexpensive chocolate available, the budget in the 3:5C condition was contracted relative to baseline to allow the re-selection of the commodity bundle chosen under baseline price levels. Under this condition, we found a trend towards higher choice proportions for chocolate in early trials, which, however, did not survive Bonferroni correction as consistently as in the 5:3C vs. 5:3 condition (Fig 5B, but see S3B Fig for the similar analysis as a function of budget). The current trial-by-trial analysis thus also supports alternative #2 mentioned in the introduction: the budget context affects choice allocations from the start of a session, over and above changes in choice distributions related to price adjustments, putatively reflecting altered relative commodity valuations. We thus interpret these differences as an income-effect on choice distributions that strongly suggests that the choices of animal consumers, like humans, follow predictions derived from demand theory.

## Discussion

We have shown here that differences in budget constraints impact the evolution of choice distributions in animal consumers as they spend their budget of nosepokes between two alternative commodities, chocolate- and vanilla-flavoured soymilk. Under conditions of equal prices, rats chose chocolate at levels above chance. Rats responded to changes in the price ratio by shifting choice allocation, in line with a substitution effect. Importantly, our design allowed us to isolate this substitution effect from a co-occurring income effect on choice allocation by measuring consumption changes during income-compensated and income-uncompensated price changes. In our design, income was compensated by adjusting the budget for price changes to the revealed preferences as exhibited through commodity bundle selection. Compensating budgets affected choice distributions in comparison to uncompensated conditions both when the preferred commodity chocolate was relatively expensive and when it was relatively inexpensive. Consequently, the budget contexts (compensated vs. uncompensated) resulted in different measures of demand elasticity (ε). Importantly, these observations held for choice sets consisting of equal proportions of budget (Fig 4) and were obvious before budgets ran out in the uncompensated conditions (Fig 5, S3 Fig), ruling out explanations according to which budget effects on demand elasticity are merely the trivial result of the number of choices considered, or selective satiation effects that might have occurred at the tail of the extended budget sessions. Rather, our findings are consistent with the hypothesis that budget context affects choice distributions directly, putatively through modifying relative valuation of the commodities already from the start of a session.

Our work replicates and extends early basic findings on budget allocation by animal consumers [4,5,10]. Using essential commodities (food pellets and water), Kagel and colleagues found inelastic demand (0 > ε > -1) in contrast to their further experiments using non-essential commodities such as root beer and Tom Collins mix (ε < -1). Our results indicate that our rat consumers’ demand for the chocolate soymilk solution was inelastic, at least over the range of prices studied here. Furthermore, chocolate elasticity was larger for uncompensated compared to compensated budget conditions, revealing the good to be ‘normal’ in economic terms. Finally, an increase in the price of chocolate resulted in an increase of vanilla consumption and vice versa (positive cross-price elasticity), indicating that these goods were treated as gross substitutes. However, it was not the case that rats always only selected the less expensive commodity, as would be expected if the commodities were perfect substitutes. The two commodities therefore function only as partial substitutes.

Our design differs from the Kagel et al. design, however, in important ways: while their animals were living in the experimental chamber (with food and water present in the non-essential commodity experiments) and could spend their budget in a 24-hour period, our animals could spend their budgets only within daily session lasting up to one hour. In the essential commodity experiment, no other supply of food or water was given, thus creating a completely closed economy. As indicated by Hursh [12], in a closed economy, animals will try to minimize differences between patterns of food intake across different response/reinforcer schedules. Operationalized as lever presses (Kagel) or nosepokes (our study), the rats essentially perform an operant response under different FR regimes according to the current price, leading to more operant responses for food if the price of food is increased. However, a feature of the Kagel study of essential commodities is that rats *need* water to digest the food pellets, barring these goods from substitution. In our study, in compliance with animal welfare regulations, rats were allowed to gain weight over days by providing an adequate food supply, while yet leaving the rats non-satiated. This design resembles an *open* economy, as opposed to a closed economy where consumers are supposed to purchases all food and drink items from their budgets, even though the sole source of chocolate and vanilla soymilk was in the context of the daily consumption sessions in the operant cages. Under such economic constraints, it could be expected that demand for the goods would be elastic, as the additional intake of these foods was non-essential to homeostasis [12]. The fact that we found inelastic demand for chocolate soymilk in an open economy suggests that the animal consumers treated it as an essential commodity, at least over the price ranges studied here, despite receiving adequate feeding outside the experimental sessions. This could be due to the substantial bias towards this good (ca. 6–10 times more chocolate than vanilla was consumed during baseline conditions), presumably reflecting the animals’ strong choice bias towards the high-sucrose chocolate-flavoured soymilk. Indeed, Hursh & Winger [6] found that normalized demand curves for more preferred commodities are less elastic, as reflected here by the more inelastic demand for chocolate as compared to vanilla. However, if the range of prices was increased, we might speculate that demand for chocolate would become more elastic if higher prices were included, especially if they could induce a preference reversal between the commodities.

We believe that the time-resolved analysis of choice distributions provides an important extension of the analyses employed in the Kagel papers: our results indicate dynamics in choice distributions that vary with budget conditions and highlight that these differences in choice distributions are not due to differences in choice set size between budget conditions, but rather are due to a difference in sequential choice structure—putatively reflecting altered relative valuation of the choice options, between budget conditions. As there are no sensory cues in the operant cage environment directly signalling the budget conditions to the rats, the current budget condition has to be experienced. In the absence of a difference in the stimulus-response-reward contingencies between sessions with or without budget compensation (e.g. 5:3C vs 5:3 with the same FR requirements), the observed differences in choice allocation suggest that rats somehow represent or at least integrate the available budget in their decision process. Interestingly, Elsmore et al. [7] found that reducing the budget available did not alter absolute choices for an essential food, but did affect choice of a non-essential commodity (heroin). Although we did not independently manipulate the available budget without also changing prices, we found, in line with the Elsmore study, that, when budgets were contracted (in the 3:5C condition), rats responded by shifting to a higher proportion of chocolate choices earlier in the session than in the 3:5 price ratio condition (which had more trials available, Fig 5B, S3 Fig). One might characterize this as a sense of urgency to consume a certain amount of chocolate before the session might run out.

An interesting feature of our data is that the rats did not allocate all choices to one (less expensive) commodity, which might have been expected for a situation in which chocolate and vanilla were perfect substitutes with different ratio requirements. Instead, the data is more akin to choice distributions found under variable interval schedules of reinforcement (i.e. a distribution reflecting the relative rate of reinforcement), that have been extensively studied within the Matching Law framework [13,14]. Why then, do our animal consumers tend to distribute their choices amongst both alternatives? It is well known that rodents have a tendency to alternate between spatial trajectories when placed in environments where different choice options are available in different spatial locations, a behavior called spontaneous alternation (reviewed in [15]) that is thought to reflect exploratory drive. In operant tasks with a probabilistic reward structure, subjects often exhibit win-stay, lose-shift strategies [16, 17]. However, even under deterministic reward contingencies, it could be worthwhile to explore the option space in an environment: after a number of choices for A, B might become progressively more attractive, perhaps due to decreasing marginal utility derived from consecutive A’s [1–3]. Indeed, naturalistic models of decision making such as those derived from Foraging Theory [18] explicitly consider the benefits of both exploitation and exploration strategies. In contrast to the marginal utility model, in this scenario, the attractiveness of the current exploitative (foreground) strategy decreases through reductions in the marginal returns of foraging time investments, for example in the case of a forager trying to gather ever more out-of-reach berries in shrubbery. When experiencing such contracting marginal return rates it pays to check the cost/benefit ratio of the alternative (background) option [19–20]. The current experiment cannot identify *why* the rats chose to distribute their budget amongst both alternatives. More importantly, spontaneous alternation alone cannot explain why choice allocation, or indeed choice sequences, would be different for different budget sets. It is tempting to speculate, however, that spontaneous alternation between the choice options in our task would be expected most for the condition in which the choice options’ costs/benefit ratios were perceived to be closest by the animal consumers, i.e. the 5:3 condition; the only condition to exhibit choice distributions not significantly different from indifference (chance) levels. Indeed, we found that the conditional probability to reselect chocolate (the inverse of alternation) after a streak of N = 1,2, or 3 chocolate choices was significantly greater in the 4:4 and 5:3C conditions as indicated by a repeated-measures ANOVA with length of chocolate chain and condition as within-subject factors: F_(2, 20)_ = 5.68, p<0.05; partial η^2^ = 0.53. Post-hoc analyses revealed that both the 4:4 and 5:3C conditions showed significantly higher conditional probabilities of reselecting chocolate than the 5:3 condition (p<0.05, LSD-corrected for multiple comparisons, 4:4 vs. 5:3C ns) with a trend to a widening of the gap with increasing sequence length (S4 Fig), suggesting again that differences in relative valuation could bias choices throughout the extent of the budget by subtly affecting reselection probabilities.

What process could drive this difference in relative valuation of the commodities between the budget conditions? Viewed from the level of the single trial, no changes in reinforcement contingencies exist between the 5:3 and 5:3C, or the 3:5 and 3:5C conditions: cost/benefits ratios are the same whether budgets are compensated or not. Any change in choice distributions between budget conditions thus must come from perceiving the choice as part of the entire budget. A tentative explanation for such an effect might lie in the difference in the costs between both commodities *relative* to the budget. In the 5:3C extended budget condition, this 2-nosepoke price difference represents a relatively smaller chunk of the entire budget than in the 5:3 condition. Conversely, in the 3:5C condition it represents a larger chunk compared to the 3:5 condition. Intuitively, it makes sense why this difference in relative price should matter: a difference of 2 nosepokes is a big deal on a tight budget, but maybe not so much on a more accommodating budget. If we combine this with the notion that our rat consumers all prefer chocolate over vanilla, as shown in the 4:4 baseline conditions, we can argue that extending the budget reduced the salience of the price differences, leading to more choices of the preferred, but expensive commodity (Fig 5A, S3A Fig). Contracting the budget, on the other hand, makes the price difference more salient, pushing consumption even further towards the cheaper, preferred commodity (Fig 5B, S3B Fig). This line of reasoning then leads to the hypothesis that contracting the budget when the preferred commodity is expensive would lead to a shift in choice distributions towards even lower consumption of the expensive commodity as the contraction increases salience of the difference, reducing purchases of the expensive commodity relative to the same price change with unchanged budgets. This suggestion remains to be investigated.

Taken together, we have shown here that rats’ subjective valuation of a liquid commodity is a function of basic preferences and/or choice biases, the commodity’s price and, importantly, budget constraints. Our results indicate that the analysis of demand using non-essential, non-addictive commodities with rats tested in an open economy is feasible and can be used to differentiate commodities based on demand elasticity. The observed non-stationarity of preferences as inferred from choice distributions within a session serves as a reminder that choice distributions should be examined across trial sets that are closely matched in time as well as in quantity. Such experimental control is ensured by employing programmed operant testing environments that impose limits on when and how often reinforcers are available. Our experimental design accommodates within-subject comparison of demand elasticity under compensated and uncompensated price changes. Such an approach allows neurobiological interventions to take place between compensated and uncompensated price changes to investigate, for example, the neuropharmacological substrates of the observed budget effect. There is ample evidence that dopamine (DA) in the Nucleus Accumbens is required for sustaining operant responding under higher effort requirements ([21], for a review see [22]). Recent advances in the field of intracranial self stimulation have been able to separately model reward sensitivity and price effects [23,24] and show that DA is specifically involved in processing rewarding effects along the price axis, perhaps by altering subjective effort costs or absolute reward intensity [25,26]. Based on these results, we hypothesize that reducing DA in the Nucleus Accumbens will make rats more sensitive to price differences, resulting in increased elasticity for the preferred commodity (cf. [22]). This hypothesis is currently being evaluated in our lab using bilateral 6-OHDA lesions.

## Supporting Information

S1 Fig Coefficient of variation decreases after session 2. To assess the variation in the choice data as a function of session number, we calculated the coefficient of variation (CV) across all sessions ran for a given session pair, per rat, per phase (6 rats * 7 phases). The panel shows the average CV and SEM. The CV for sessions 1&2 per phase is significantly different from the average of the subsequent 4 session pairs. *: paired-sample t-test *t*_(17)_ = 2.34; p = 0.03.
(EPS)

S2 Fig Individual choices for chocolate as a function of inverted price ratios (for cross-price elasticity calculations). Log-transformed choices for chocolate (left panels) or vanilla (right panels) on the y-axis are plotted as a function of the log-transformed price ratio for vanilla over chocolate (left panels) or chocolate over vanilla (right panels), per rat, for uncompensated budgets (top panels) and compensated budgets (bottom panels), respectively.
(EPS)

S3 Fig Time-resolved proportion of choice for chocolate as a function of budget spent. As in Fig 5A and 5B, but now showing the time-resolved fraction of choice for chocolate evaluated over a sliding 30-nosepoke budget window. Plot conventions as in Fig 5; Purple bar: Difference in Z-value > 1.96 (p<0.05, Bonferroni-corrected for multiple comparisons).
(EPS)

S4 Fig Conditional probability of re-selecting chocolate as a function of length of chocolate choice chain. The conditional probability of reselecting chocolate as a function of 1,2 or 3 recent chocolate choices is plotted for the averaged baseline condition (4:4, green), the compensated expensive chocolate condition (5:3C, yellow) and the uncompensated expensive chocolate condition (5:3, brown). The conditional probability is significantly different between conditions: rmANOVA with length of chocolate chain and condition as within-subject factors: F_(2, 20)_ = 5.68, p<0.05; partial η^2^ = 0.53. Post-hoc analyses revealed that both the 4:4 and 5:3C conditions showed significantly higher conditional probabilities of reselecting chocolate than the 5:3 condition (p<0.05, LSD-corrected for multiple comparisons, 4:4 vs. 5:3C ns).
(EPS)

S1 Table Shaping steps leading up to the final experiment settings. (DOCX)
