# Supplementary material for: Budget Constraints Affect Male Rats’ Choices between Differently Priced Commodities
Source: PLoS One. 2015 Jun 8;10(6):e0129581. doi: 10.1371/journal.pone.0129581 (PMC4460023; doi:10.1371/journal.pone.0129581)
Supplement: S1 Table — (DOCX) [file pone.0129581.s005.docx]

TABLE S1

For:

Budget constraints affect male rats’ preferences for differently priced rewards

Short title: Budget effects on rat decision-making

M. van Wingerden^1^*^¶^, C. Marx^1¶^ and T. Kalenscher^1^

^1^Comparative Psychology, Institute for Experimental Psychology, Heinrich-Heine University, Universitaetsstrasse 1, D-40225 Düsseldorf, Germany

*Correspondence at: Marijn.Wingerden@hhu.de

^¶^These authors contributed equally to this work

*Table 1: Shaping steps leading up to the final experiment settings.*

| ***Step*** | ***Central nosepoke*** | ***Lateral nosepoke*** | ***Delay to Reward*** | ***FR*** | ***Introduced component*** | ***Criterion to promote*** |
| --- | --- | --- | --- | --- | --- | --- |
| 0 | >50 ms in any unit | | - | FR1 | initiation nosepoke | 40 correct trials |
| 1 | >50 ms | >50 ms | - | FR1 | lateral nosepoke | 40 correct trials |
| 2 | >400ms | >100ms | 10ms | FR1 | bottle access delay | 40 correct trials |
| 3 | >400ms | >100ms | 10ms | FR1 | Forced and Free choice trials | 52 free correct trials, 8 forced correct trials |
| 4 | >500ms | >100ms | 100ms | FR2 | Increasing Fixed Ratio | 52 free correct trials, 8 forced correct trials |
| 5-7 | >500ms | >100ms | 100ms | FR2 –FR4 | Increasing Fixed Ratio; introduction of budget | 100% of budget spent in 5 sessions |
| Exp | >500ms | >100ms | 100ms | FR3- FR5 | Budget constraints, different price ratios |  |

*FR: Fixed Ratio*
